# Solubility of Two Root-End Filling Materials over Different Time Periods in Synthetic Tissue Fluid: a Comparative Study

**Published:** 2015-09

**Authors:** Nooshin Sadat Shojaee, Safoora Sahebi, Elahe Karami, Fereshte Sobhnamayan

**Affiliations:** 1 Dept. of Endodontic, School of Dentistry, Shiraz University of Medical Sciences, Shiraz, Iran.; 2 Student, School of Dentistry, Shiraz University of Medical Sciences, Shiraz, Iran.

**Keywords:** Calcium Enriched Mixture, CEM, Mineral Trioxide Aggregate, MTA, Solubility

## Abstract

**Statement of the Problem:**

Insolubility is an important criterion for an ideal root-end filling material to both prevent any microleakage between the root canal and the periradicular space and provide sealing ability.

**Purpose:**

Many recent studies have shown that mineral trioxide aggregate (MTA) and calcium-enriched mixture (CEM) have acceptable sealing ability. The purpose of this *in vitro* study was to evaluate the solubility of these root-end filling materials.

**Materials and Method:**

Forty stainless steel ring moulds with an internal diameter of 10±1 mm and a height of 2±0.1 mm were selected. Samples of MTA and CEM were mixed according to the manufacturer’s instructions and inserted into the moulds. The specimens were divided into 4 experimental groups and kept in synthetic tissue fluid (STF) for 2 different time periods (7 and 28 days). The control group contained 8 empty rings. The moulds’ weights were recorded before and after immersion in STF. The changes in the weight of the samples were measured and compared using a two- way ANOVA test at a significance level of 5%. Specimens were evaluated with scanning electron microscopy (SEM) at a magnification of 500×.

**Results:**

There was no significant difference in weight changes between MTA and CEM samples (*p*> 0.05).

**Conclusion:**

MTA and CEM have similar solubility in STF in different time periods.

## Introduction

Root-end filling materials are used to provide an apical seal and prevent apical leakage of irritants into the periradicular tissue.[1] The characteristics of an ideal root-end filling material would include biocompability,[2] sealing ability[3] and cementogenic activity.[4] One of the most important characteristics of an ideal material is insolubility in various acids, enzymes, and fluids in the oral cavity to provide sealing ability and block the migration of bacteria and their products into the periradicular tissues.[5] Therefore, due to permanent contact with fluids in the oral environment, the insolubility of materials used in the oral cavity is an important concern.[6]

A number of materials have been used for this purpose. Mineral trioxide aggregate (MTA) was introduced in 1993 and is an acceptable material.[7] MTA consists of hydrophilic particles that set in the aqueous environment.[8] The sealing ability of MTA has been reported to be superior to that of amalgam, IRM and Super EBA.[9-11] MTA has bioactive properties, can release calcium and expands during setting.[12] It induces proliferation, and not apoptosis, of pulp cells *in vitro*. This material has different clinical applications[13] such as use as a root-end filling material,[14] apical plug[15] and repair of root perforations.[16-17]

A novel endodontic cement was developed by Asgary *et al.* in 2006.[18] The calcium enriched material (CEM) (US patent 8105086 B2) is biocompatible,[19] easy to handle and set in an aqueous environment,[20] able to stimulate hard tissue healing[21] and it provides an effective seal when used as a root-end filling material.[22] CEM has good sealing ability comparable to MTA, and its antibacterial effect is comparable to calcium hydroxide and greater than MTA.[22] Some recent studies using different techniques, such as dye penetration[18, 22-23] and the fluid filtration method,[24] have shown that CEM exhibits similar sealing properties to MTA. Saghiri *et al.* evaluated the solubility of MTA in various media. They used synthetic tissue fluid (STF) to produce similar clinical conditions due to its similar composition to dentinal fluid. They concluded that although MTA dissolves faster in deionised water, its solubility in both media was acceptable.[5]

Additional studies have evaluated the solubility of root-end filling materials.[5-6,25] Fridland and Rosado evaluated the solubility of gray MTA using different water-to-powder ratios. They showed that solubility increased when the water-to-powder ratio increased.[6] Poggio *et al.* reported that all tested materials included Pro Root MTA, IRM and Superseal demonstrated a weight loss of less than 3% which fulfilled the requirements of International Standard Organization (ISO) 6876.[8]

As mentioned previously, there are many studies that have shown that MTA and CEM have similar properties and applications.[22-24] To the best of authors’ knowledge, no study has yet investigated the solubility of CEM. Therefore this study designed to compare the solubility of these materials and the weight changes of MTA and CEM over two different time periods in STF

## Materials and Method

Two different root-end filling materials (MTA, CEM) were included in this experimental study. The solubility of these materials was evaluated in accordance with the International Standard Organization (ISO) 6876 method.[26] 40 stainless steel ring moulds with an internal diameter of 10±1mm and a height of 2±0.1mm were selected and cleaned in an ultrasonic bath of detergent for 15 min to remove grinding chips. All the rings were then weighed and each mould was identified with a code. Each ring was weighed twice on a precision scale with 0.01g accuracy (AND EK 300i) before and after filling the moulds and their weights were recorded by the predetermined codes. Angelus MTA (Angelus Soluc*x*o˜es Odontolo´gicas; Londrina, Brazil) and CEM (Biunique Dent; Tehran, Iran) were mixed by a single operator according to the manufacturer’s instructions (1 gram powder mixed with 0.33 ml distilled water) and care was taken to prevent air bubble entrapments. The cement was condensed using a spatula and then allowed to cure in the stainless steel moulds. Flat glass plates with dimensions larger than the ring moulds were used to flatten the surface of the specimens and remove cement flushes.

Wet gauze was put on the upper and lower surfaces of the cements to produce 95 % humidity and all specimens were placed in the incubator at 37^o^C for a time period 50% longer than the complete setting time stated by the manufacturer to being set completely.

The samples of MTA and CEM were divided into 2 separate groups and immersed in STF for 2 different time periods. The STF is a phosphate buffer saline solution with pH=7.2 and composed of 11.80g Na_2_HPO_4_, 80.0g NaCl and 2.0g KCl in 10L of H_2_O. The specimen were immersed in a container with 100 ml of this fluid and then sealed with plastic wrap and placed in the incubator at 37°C. The STF was refreshed every 3 days.

**Figure 1:**
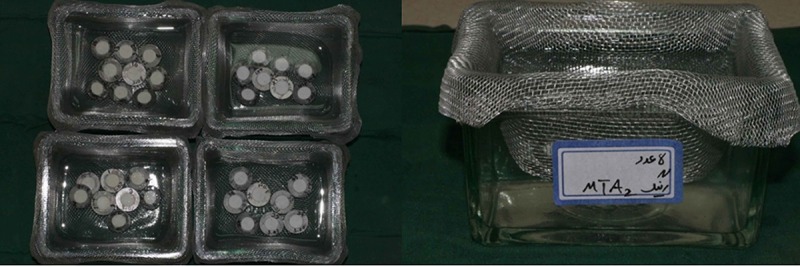
The containers with experimental ring moulds immersed in STF. Each mould has a code to record its weight before and after the immersion time period.

Figure 1 shows the experimental groups which were arranged as group 1; 8 rings filled with MTA and kept in STF for 7 days, group 2; 8 rings filled with MTA and kept in STF for 28 days, group 3; 8 rings filled with CEM and kept in STF for 7 days, group 4; 8 rings filled with CEM and kept in STF for 28 days, and group 5; (negative control) with 8 empty rings.

The samples were removed from the incubator after the predetermined time period. Each mould was washed carefully with distilled water, dried with drier paper placed in the oven (at 105°C with no humidity) for 24 hours and then cooled down in the same desiccator. 

The weight of the rings was measured, the weight differences were calculated and the percentage of weight changes was recorded. One specimen from experimental groups 2 and 4 was selected to evaluate with scanning electron microscopy (SEM) at a magnification of 500×.The weight changes of the experimental materials were analysed by the two-way analysis of variance (ANOVA) test (SPSS, version 20) at a significance level of 5 % (α = 0.05).

## Results

There was no change in the weights of the empty rings in the control group after immersion in STF through the two time periods. MTA and CEM weights changed significantly in both experimental intervals. MTAʼs weight increased by 0.5 % and 2 % in 7 and 28 days, respectively, and CEM’s weight increased by 0.6 % and 3% in 7 and 28 days, respectively, but there was no significant difference between the MTA and CEM groups )*p*> 0.05) (Table 1).

**Table 1:** Changes in solubility values (percent mean weight changes) of the MTA and CEM groups stored in STF. The mean and standard deviation values were for MTA and CEM at 7 and 28 days.

**Solubility** **Experimental material**		**Mean**	**Standard ** **deviation**
7 days	MTA CEM	0.5%	2.6%
		0.62%	0. 518%
28 days	MTA CEM	2.12%	0.354%
		3%	0.926%

**Figure 2:**
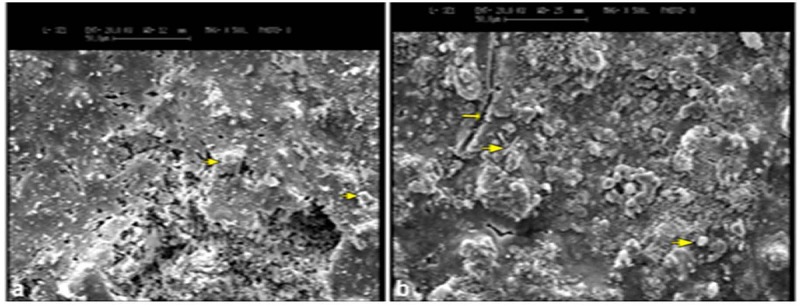
: SEM image of MTA at 28 days with amorphous crystals (small arrows) b: SEM image of CEM surface immersed in STF at 28 days with porous capillaries (small arrow) and small, needle-like and amorphous crystals (large arrows) scattered in some areas (original magnification, 500×).

There was also no statistically significant difference between time intervals in the MTA and CEM groups (*p*> 0.05). The pictures obtained from the SEM evaluation showed the MTA and CEM specimens after the different time periods. Evaluation of the MTA and CEM surfaces showed numerous amorphous crystals with the presence of voids and porosities (Figure 2).

## Discussion

Insolubility is an important factor for a desirable root- end filling material. In clinical applications like endodontic surgery, the material is in direct contact with-various acids, enzymes, and fluids. Therefore, good sealing ability has critical importance. The material cannot attach unless it is insoluble in natural oral fluids.[27-28]

In the present study, we soaked the samples in STF which is a phosphate buffer saline solution with a composition similar to biological fluids, such as interstitial fluids and dentinal fluid.[29] Saghiri *et al.* suggested this property of STF makes it a good alternative media for materials used in solubility studies.[5]

MTA is a bioactive material; therefore, in an oral environment or in contact with STF, it dissolves and releases its major cationic components. This reaction leads to the production of hydroxyapatite (HA) on its surface and perhaps causes expansion and an increase in weight.[29] Sarkar *et al.*[29] showed that calcium (Ca^2+^) is the most dominant ion released from MTA in contact with STF. Because this ion is sparingly soluble in STF, it causes the HA to precipitate. Since STF is a phosphate buffer saline solution, it contains major cationic constituents such as phosphate ions. Asgary *et al.*[30] evaluated the effect of a phosphate buffer solution on the topography of MTA and CEM. Their SEM evaluation showed that HA crystals were formed over the both cements when in contact with phosphate buffer solution, which contains phosphate ions. An *in vitro* study by Amini Ghazvini *et al.*[31] showed that CEM released a higher concentration of Ca^2+^ ions in different time intervals than did MTA. HA precipitation was promoted by contact with the Ca^2+^ ions released from the cement.

In an SEM study, Salem Milani *et al.*[32] reported that samples exposed to STF had an irregular crystalline microstructure. Hexagonal and plate-like crystals with well-defined borders and amorphous crystals were observed on the MTA samples exposed to STF. The current study obtained SEM micrographs of the specimens to evaluate the surface structure, which showed the voids and porosities on the surfaces of both MTA and CEM after storage in STF. Some amorphous and needle-like crystals were observed on the surfaces of both MTA and CEM that were in contact with STF (Figure 1).

Many studies have shown that MTA and CEM exhibit similar sealing properties.[22-24] As previously mentioned, this can be explained by the dissolution of CEM in contact with STF that leads to HA precipitation in the same manner as MTA.

In an *in vitro* study, Saghiri *et al.*[5] compared the solubility of MTA in different media. They found that MTA was less soluble in STF than in distilled water, perhaps due to the higher concentration gradients of minerals and lower penetration of STF into the cement.[5] In contrast to these results, the current study experienced that both cements were similarly insoluble in STF. Indeed, both cements gained weight. Considering the lower solubility of MTA in STF, Saghiri *et al.*[5] suggested that STF was a better media than distilled water for evaluating the solubility of dental materials. Therefore in this study STF was selected as the preferred media.

In the present study, CEM and MTA similarly gained weight when they were in contact with STF during the experimental time periods. In contrast, an *in vitro* study by Bodanezi *et al.*[25] showed that both MTA and Portland cement had weight gain in the first hours, followed by some weight loss thereafter. They used distilled water as the media. In our study, all of the samples’ weights were increased in the four experimental groups. This difference can be explained by the different media used in each study. We used STF that has a similar composition to the natural aqueous environment in dentin, perhaps leading to more chemical reactions and an increase in the production of HA. Thus, the material’s weight would increase in contact with this liquid when compared with distilled water.

## Conclusion

Under the conditions of this *in vitro* study it was concluded that MTA and CEM gained weight in STF during the experimental time period, but this was not significant. Therefore, MTA and CEM are suggested to be used as root-end filling materials.
